# Expression Profiling the Temperature-Dependent Amphibian Response to Infection by *Batrachochytrium dendrobatidis*

**DOI:** 10.1371/journal.pone.0008408

**Published:** 2009-12-22

**Authors:** Laia Ribas, Ming-Shi Li, Benjamin J. Doddington, Jacques Robert, Judith A. Seidel, J. Simon Kroll, Lyle B. Zimmerman, Nicholas C. Grassly, Trenton W. J. Garner, Matthew C. Fisher

**Affiliations:** 1 Department of Infectious Disease Epidemiology, Imperial College London, United Kingdom; 2 Department of Paediatrics, Imperial College London, London, United Kingdom; 3 University of Rochester Medical Centre, Rochester, New York, United States of America; 4 Developmental Biology, National Institute for Medical Research, London, United Kingdom; 5 Institute of Zoology, Regent's Park, London, United Kingdom; Universidade de Sao Paulo, Brazil

## Abstract

Amphibians are experiencing a panzootic of unprecedented proportions caused by the emergence of *Batrachochytrium dendrobatidis* (*Bd*). However, all species are not equally at risk of infection, and risk is further modified by environmental variables, specifically temperature. In order to understand how, and when, hosts mount a response to *Bd* we analysed infection dynamics and patterns of gene expression in the model amphibian species *Silurana (Xenopus) tropicalis*. Mathematical modelling of infection dynamics demonstrate the existence of a temperature-dependent protective response that is largely independent of the intrinsic growth-rate of *Bd*. Using temporal expression-profiling by microarrays and qRT-PCR, we characterise this response in the main amphibian lymphoid tissue, the spleen. We demonstrate that clearance of *Bd* at the host-optimal temperature is not clearly associated with an adaptive immune response, but rather is correlated with the induction of components of host innate immunity including the expression of genes that are associated with the production of the antimicrobial skin peptide preprocareulein (PPCP) as well as inflammatory responses. We find that adaptive immunity appears to be lacking at host-optimal temperatures. This suggests that either *Bd* does not stimulate, or suppresses, adaptive immunity, or that trade-offs exist between innate and adaptive limbs of the amphibian immune system. At cold temperatures, *S. tropicalis* loses the ability to mount a PPCP-based innate response, and instead manifests a more pronounced inflammatory reaction that is characterised by the production of proteases and higher pathogen burdens. This study demonstrates the temperature-dependency of the amphibian response to infection by *Bd* and indicates the influence that changing climates may exert on the ectothermic host response to pathogens.

## Introduction

Amphibians are experiencing a panzootic of unprecedented proportions caused by the emergence of *Batrachochytrium dendrobatidis* (*Bd*) [1], [2], [3], [4]. While anthropogenic spread of *Bd* appears to be driving the global distribution of the pathogen [5], disease and declines are modulated by a complex interaction between environment, host and pathogen-specific factors [6], [7]. Research has identified temperature as a key factor in predicting the spatial occurrence of *Bd*-related declines [8], [9], and longer-term climate trends appear to drive these patterns of disease by amplifying the growth of *Bd* at its thermal optimum [6], [7]. However, this model ignores the potential contribution of host immunity. While it is known that amphibian antimicrobial peptides (AMPs) correlate with species survival against *Bd* [10], [11], it is not known if amphibians mount a specific response against *Bd*, or to what extent any host response is temperature-dependent. Amphibians are ectotherms, and seasonal temperature variability exerts strong direct effects on their immune system [12]. As a consequence, the susceptibility of amphibians to pathogens is expected to vary between seasons. Across longer temporal scales, global warming is negatively affecting survival of even widespread habitat-generalists such as the common toad *Bufo bufo* [13]. Therefore, the outcome of infection by *Bd* is likely to be exacerbated by synergies between short-term temperature-dependent effects on the immunocompetence of animals and longer-term physiological stressors.

**Figure 1:**
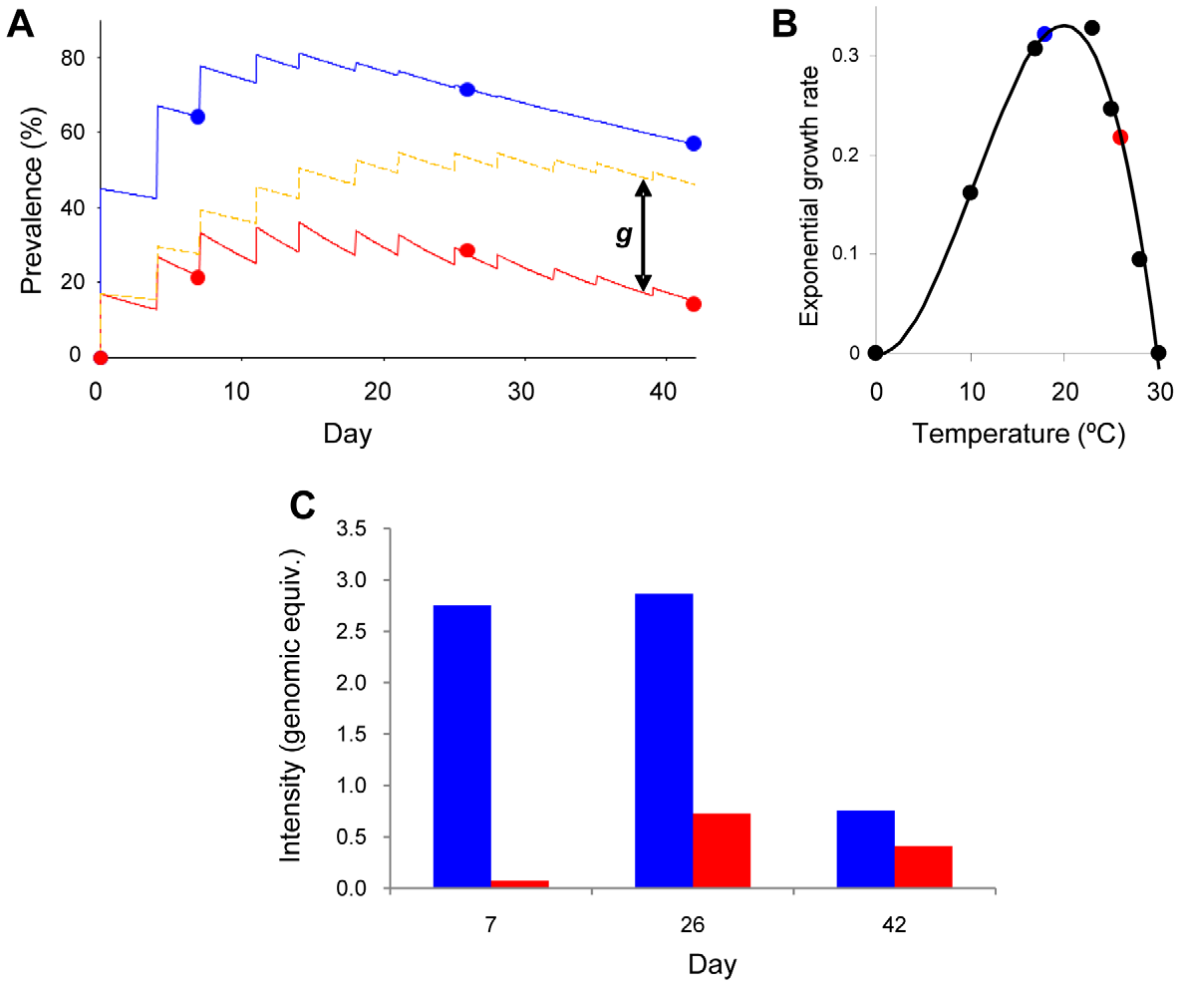
Experimental infection and model outputs. **A.** Observed prevalence data for iCT animals (blue circles) and modelled prevalence data (blue line, RSS (residual sum of squares)  = 0.112), and observed prevalence data for iWT animals (red circles) and modelled prevalence data (solid red line, RSS = 1.99). Dotted orange line shows modelled iWT prevalence data where an identical host response to iCT animals has been assumed. *g* means the gap between modelled prevalence in iWT with and without differing host response due to temperature. Modelling prevalence without taking into account a differing host response does not fit the observed data well (RSS = 1611). **B.** Third order polynomial fitted to measurements of temperature against daily exponential growth rate of *Bd* in culture from [14] (y = -0.00008639x^3^ + 0.002612x^2^ – 0.001124x – 0.0004419). Blue circle indicates *Bd* growth rate at the temperature of iCT animals, and red circle indicates *Bd* growth rate at the temperature of iWT animals. **C.** The intensity of infection measured by qPCR as zoospore genomic equivalents.

Here, we describe the use of mathematical models and post-genomic technologies to identify the pathogen-associated gene expression profiles that a model amphibian species, *Silurana* (formerly *Xenopus*) *tropicalis*, exhibits in response to infection by *Bd* at different temperatures. We focus on defining the temporal dynamics of infection at two infection time-points, days 7 and 42, and at two temperatures; these temperatures were chosen to reflect the ‘tropical’ environmental norm for *S. tropicalis*, 26°C, and a lower, non-optimal ‘temperate’ temperature, 18°C. By using these two temperatures, we shifted the animals' environment towards the growth optimum for *Bd* (*in vitro* between 17°C and 25°C (Figure 1) [14]. In order to quantify the importance of host immunity and *Bd* growth-rate at these two different temperatures, we profiled the prevalence and intensity of *Bd* burdens in frogs over a 42-day infectious time course. By using a simple mathematical model, we estimated the contributions of host-response and *Bd-*growth to the observed infectious outcomes. In tandem, we investigated variation in the host response by profiling time and temperature-dependent global gene expression profiles in the main lymphopoietic tissues in frogs, the spleen by using microarrays and qRT-PCR transcriptomic strategies. Finally, we compared our data with the recent publication of the *S. tropicalis* transcriptome-response to *Bd* exposure at cool (18°C) temperatures [15].

## Results and Discussion

### *Bd* Infection Dynamics

Quantitative *Bd*-specific PCR shows that the prevalence and intensity of infection peaked in animals at each temperature between days 7 and 26 (Figure 1A & C). This suggested that the frogs were mounting an anti-*Bd* response in both groups, as animals were exposed to high infections (∼10^6^ zoospores per procedure) throughout the course of the experiment; in the absence of a host response levels of infection should climb throughout the lifetime of the experiment. There was a higher prevalence and intensity of infection in iCT (infected Cold Temperature group) compared to iWT (infected Warm Temperature group) frogs throughout the experiment (Figure 1). Fitting a mathematical model of the infection process (Figure 1A) showed that animals in the iWT treatment had approximately a third of the chance of infection per procedure compared with those in the iCT treatment (*β_iWT_* = 0.170 and *β_iCT_* = 0.451) and, once infected, iWT animals recovered almost five times as fast (γ*_iWT_* = 0.071 and γ*_iCT_* = 0.015). After accounting for the greater *Bd* growth rate at the cooler temperatures, the fit of our model was consistent with iWT frogs clearing zoospores approximately twice as fast as iCT frogs (*c_ iWT_* = 1.322 and *c_ iCT_* = 0.646). Modelling prevalence assuming an identical host response in iWT animals as in iCT animals does not fit the observed data well (Figure 1). This finding suggests that iWT frogs mount a more effective immune response to *Bd* than do iCT frogs.

### Overview of Patterns of Differential Gene-Expression

**Figure 2:**
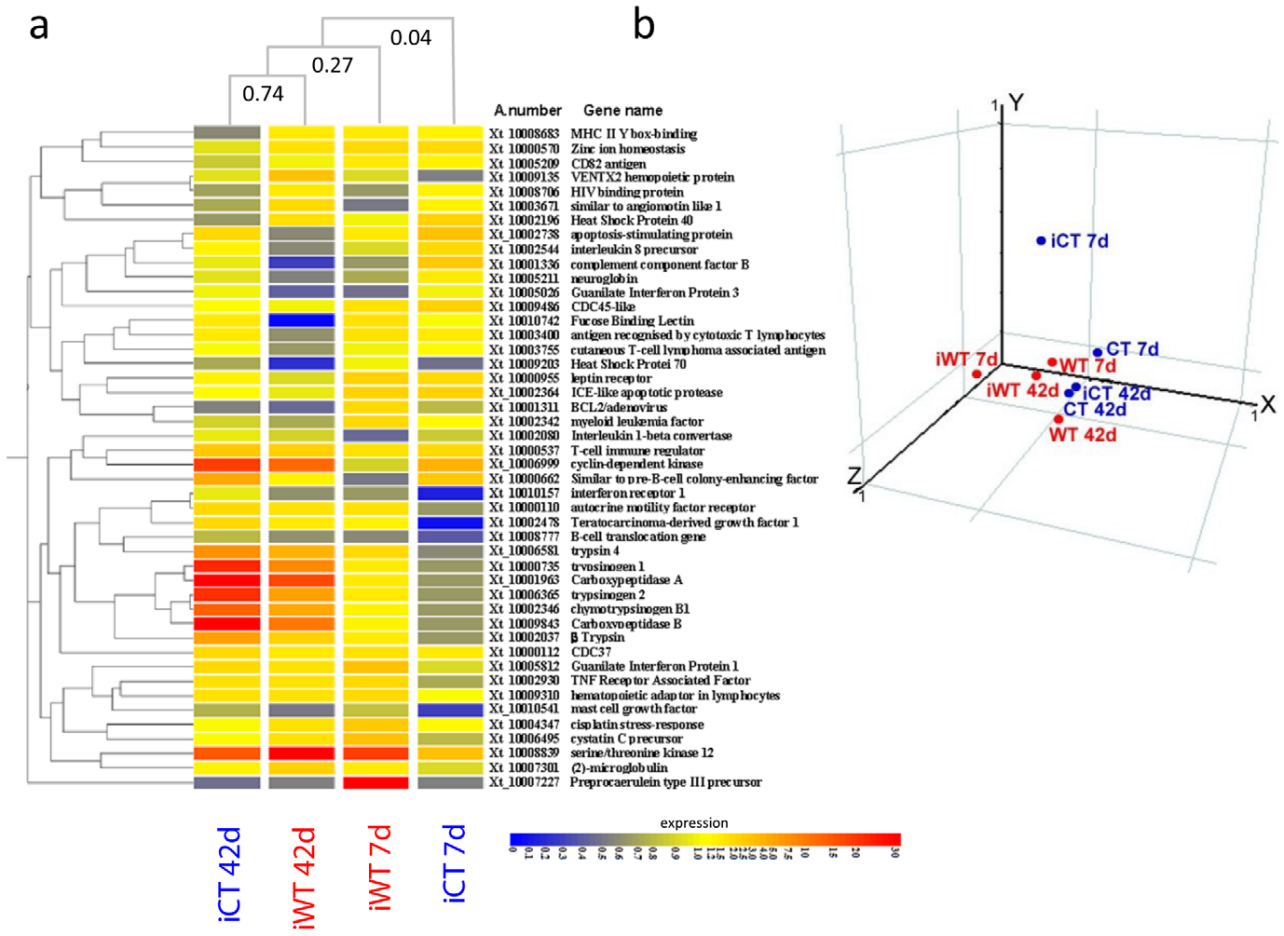
Microarray results in spleen of *S. tropicalis Bd* infected. **A.** Expression of the top 50 genes obtained from microarrays with known or putative immune-function as shown by two-way gene clustering and clustering analysis (GeneSpring Software). The colour scale shows the fold changes above normalised controls. **B.** Principal Component Analysis (PCA) showing that *S. tropicalis* frogs held at cold temperatures (iCT) have the most diverse transcriptional profile during the first stage (7 days) of infection. Axis: X = PCA Component 1 (24.99% variance); Y = PCA Component 2 (18.12% variance); Z = PCA Component 3 (14.95% variance).

To identify the molecular basis of the host response to *Bd* infection, we used microarrays to profile gene expression in the cold and warm infected cohorts at days 7 and 42 of infection (see Figure S1 for the experimental design). We focused on the spleen which, in the absence of lymph nodes, is the major lymphoid organ in *S. tropicalis*. This profiling revealed that there was a robust infection-specific aspect to the global induction of gene expression following exposure to *Bd*, and this response was both temperature- and time-dependent. From a total of 10,878 probes, following normalization to the controls, 434 genes for the iCT and 492 genes for the iWT groups were differentially regulated throughout the experiment. On day 7, frogs had received two exposures to *Bd* with the result that the ratio of significantly up-regulated:down-regulated genes (the “up∶down ratio”) in iCT frogs was 133∶95, compared to a ratio in iWT frogs of 96∶88. By day 42, after twelve successive exposures to *Bd*, iCT frogs showed an up∶down ratio of 105∶81 compared to 71∶121 for iWT frogs. Within these differentially expressed groups of genes, iWT frogs expressed significantly (Student *t*-test: *p*<0.01) higher numbers of genes with a fold change >30 (2.3%) and 2.5–1.5 (74%) relative to iCT frogs where fold change ranged between >30 (1.83%) and 2.5–1.5 (56.5%). There was little overlap in expression profiles between these two time-points within groups, even when the stringency of the statistical analysis was reduced to *p*<0.05; here iCT frogs shared 3.30% of genes between days 7 and 42 and iWT frogs shared 2.62%. This finding shows that both infected groups are changing their expression profiles during the intervening 35 day period to a large degree. Temperature-independent genes that were shared between iCT and iWT groups represented 4.76% of the total expression profiles. Differentially regulated genes (>1.5 fold and *p*<0.01) in *Bd* infected groups compared against controls are summarized in Table S1. Principal component analysis (PCA) of gene expression in the spleen (Figure 2B) showed that early (day 7) iCT frogs contributed the most diverse patterns of gene-expression of any group, as well as showing the highest prevalence and intensities of infection (Figure 1). These animals were in ‘double jeopardy’ because they faced both a non-optimal temperature [16], [17] and infection by *Bd* that is growing close to, or at, its environmental optimum [14], [18].

### Differentially-Expressed Genes in ‘Warm Infected’ Frogs

**Figure 3:**
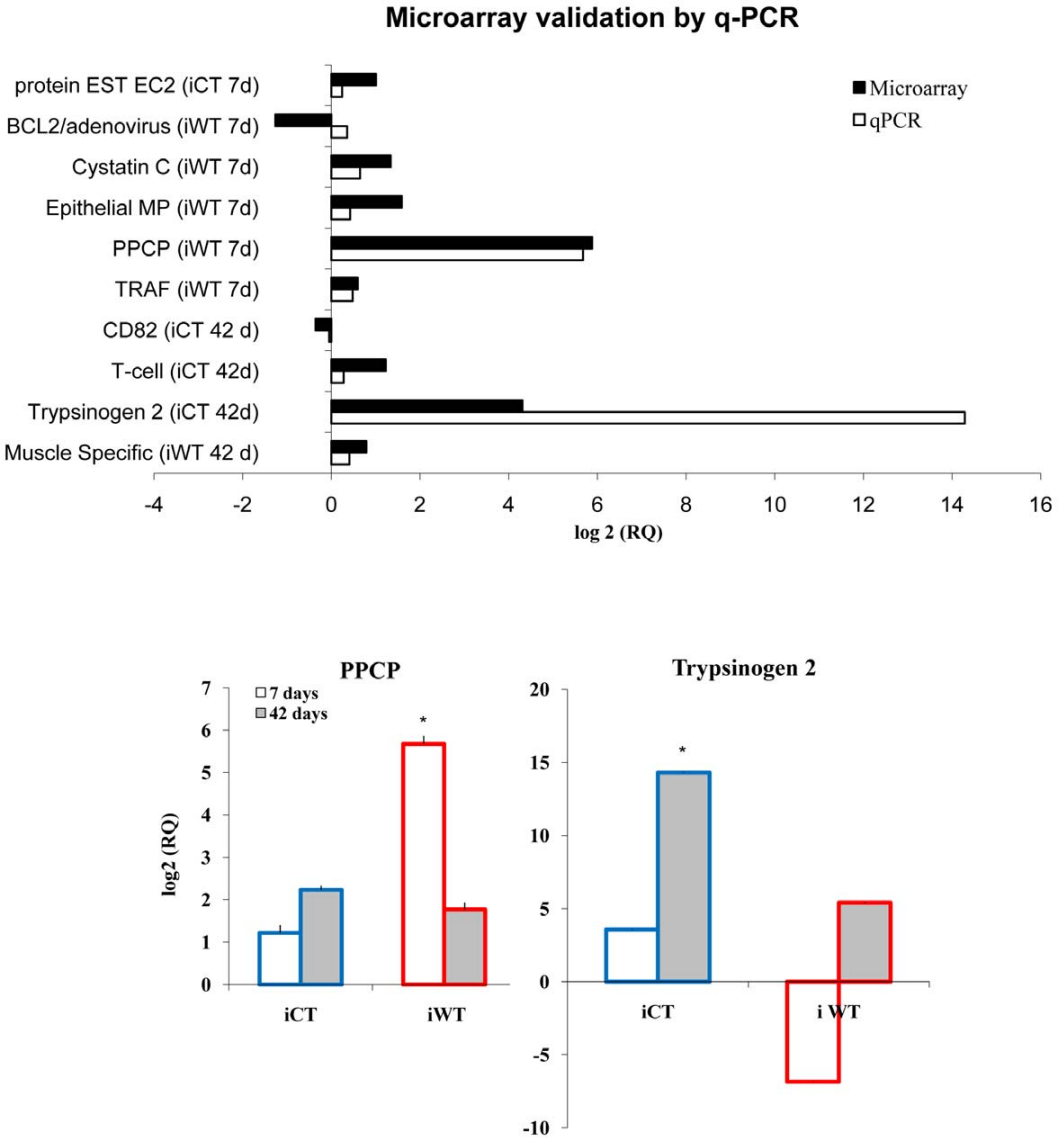
Microarray validation by quantitative RT-PCR for each of the 52 experimental frogs using spleen RNA. For qRT-PCR analysis, samples were analysed in triplicates for each individual (control and infected). A. Microarray validation by qRT-PCR amplification for a subset of 10 genes with known or suspected roles in immunity. Black bars  =  microarray data (pooled samples); White bars  =  qRT-PCR validation data (individual samples). Data is expressed by log_2_ Relative Quantification versus the control group.. B. Further transcriptomic analysis by qRT-PCR of the expression of PPCP and trypsinogen 2 genes individually tested for all frogs (*n* = 52). Data is expressed by log_2_ Relative Quantification versus the control group (iWT7/cWT7, iWT42/cWT42, iCT7/cCT7 and iCT42/cCT42) ± Standard Error. * Indicates significance by Student t-test (*p*<0.05) compared to the control group at each time point.

Strikingly, the most highly expressed gene in the spleen of iWT frogs 7 days after *Bd* exposure was the AMP preprocaerulein type III precursor (PPCP; Figure 2, 3). Microarray analysis showed that this gene was found to be expressed 59 fold change above controls in spleen (Table S1, Figure 3A). The decapeptide caerulein is a principal constituent of the skin secretion of a variety of amphibian species [19], [20], [21] and is the most potent AMP to be secreted by *S. tropicalis*, with a demonstrated antimicrobial activity against 22 microbial species including Gram-positive bacteria and the fungus *Candida albicans* [22]. The strong up regulation of the differential PPCP expression observed by the microarrays was further validated by qRT-PCR individually for the spleens of all 52 experimental frogs (WT and CT groups; Figure 3B). Interestingly, expression of PPCP was not detected in spleen, or at low levels, in the control group, and there was no significant induction of this gene in any of the CT groups. qRT-PCR showed that PPCP expression levels were up to 71-fold upregulated in day 7 iWT frogs, confirming our microarray results. These results suggest that PPCP is specifically upregulated by *S. tropicalis* in response to *Bd* infection, and that induction only occurred at temperatures that are optimal for the host. Production of PPCP-protein has not previously been observed from tissues other than granular skin glands [23], however further analysis using qRT-PCR of skin-tissue showed no clear evidence of PPCP RNA production (Ribas, unpub. obs.). Furthermore, the recent publication by Rosenblum *et al*. [15] of the transcriptomic response in the liver and skin of infected *S. tropicalis* by *Bd* at 18°C also did not find any expression of AMPs in either of these tissues. Together, these findings raise the interesting and potentially important hypothesis that the spleen could be involved in manufacturing and the subsequent trafficking of this peptide following *Bd* infection as a first response of the innate system, and that this only occurs early on in infection, and at host-optimal temperatures. However, further work is needed to investigate this hypothesis, including an investigation of the temporal nature of AMP secretion and it's efficacy in the skin against *Bd*, following infection.

Within the iWT cohort, levels of expression of PPCP had reduced to background by day 42 (Figure 3B) showing a strong temporal dependency in the expression of this gene. The production of AMPs is induced following exposure to various pathogens in other amphibian species, such as *Rana esculenta* [24], [25]. Pathogen-specific induction of AMPs has been demonstrated for this species, where it was shown that it only synthesises AMPs when microbes (bacteria or fungi) are present [25]. The reduction in PPCP production over time that we observe is possibly correlated with the recurrent nature of our infection regime; frogs were challenged every 4 days with over 10^6^ zoospores and such high levels of infectious assault are likely to exhaust the capacity of these frogs to synthesise, traffic and discharge, PPCP [26]. In addition to PPCP, a second AMP, the Cytastin C precursor peptide, was found to be upregulated by 2.6 fold (Table S1, Figure 2A, 3*a*) increasing the evidence of the key role of AMPs in protection of amphibians from pathogens [27]. However, as for the PPCP results described above, it is unclear whether increased expression of this protein in the spleen represents *de novo* synthesis of this AMP, or rather its presence in the spleen is a consequence of the system-wide trafficking of this precursor peptide.

It is estimated that *X. tropicalis* genome has around 28,000 genes, therefore the Operon v1.0 *S. tropicalis* microarray that we use here represents a subset of around 40% of these genes and lacks probes for many of the immune genes involved in the anti-pathogen response. Nevertheless, cluster analysis identified a number of genes with homology to vertebrate genes with known roles in immunity. We identified 50 genes with known immune-associated function, and demonstrate the upregulation of several other genes with suspected roles in vertebrate innate immunity (Figure 2A). Notably, genes encoding cell-surface receptors were upregulated over the first stages of infection, indicating that pathways involved in innate-pathogen recognition are rapidly activated (Figure 2A, 3A). For example, tumour necrosis factor associated factor (TRAF, 1.5 fold) which interacts directly or indirectly with members of the TNFR superfamily [28], fucose binding lectin (2.3 fold) and leptin receptor (2.1 fold) were rapidly induced after two exposures of *Bd*. Subsequently, a down regulation of these pathogen-recognition genes were observed at the end of experiment (fucose binding lectin, -36.7 fold). Activation of other inflammatory markers, guanylate binding protein interferon inducible (1,44 fold), calcineurin interleukin (IL) 2 inducible (1,33 fold) and trypsinogen genes, were also observed (Figure 2, Table S1). Expression of these trypsinogen genes were significantly increased after 42 days of exposure, although this effect was not as strong as in animals infected at cold temperatures (Figure 2 and 3B, see below). Genes responsible for hemopoietic stem cell maintenance such as VENTX2 (2.6 fold) and hematopoietic SH2 protein adaptor (1.5 to 26 fold) were also increased at 7 days of infection, suggesting that hematopoietic cell development in the spleen is upregulated as a result of infection [29].

In marked contrast to the upregulation of genes involved in innate immunity, the expression of several genes with roles in the generation of an adaptive immune response were downregulated in the spleen over the experiment. Such genes include the B-cell translocation gene (−1.88 fold, day 7) and the cutaneous T-cell lymphoma associated antigen (−1.69 fold, day 42). The complement factor B, an element of the complement system which bridges the innate and adaptive immunity with a critical importance for the generation of a potent humoral response [30] was also inhibited throughout the infection in iWT frogs (−1.63 fold, day 7 and −3.33 fold, day 42; Table S1, Figure 2A). Our results are in concordance with the findings of Rosenblum *et al*. [15] where five complement pathway genes were shown to decrease their expression in *S. tropicalis* liver after *Bd* infection [15]. In this study, these authors raise the hypothesis that a lack of evidence for an adaptive immune response to *Bd* both in liver and skin tissues is suggestive of a more complex, perhaps inhibitive, effect of *Bd* on the immune system. Although more needs to be done to understand the role of specific-immunity in combating *Bd* infection, our experimental results suggest a central role of the amphibian spleen as an effector of the innate immune system, and support the conclusion that there is little evidence for an active adaptive immune-reaction.

### Differentially-Expressed Genes in ‘Cold Infected’ Frogs

Microarray data showed that expression of the AMP PPCP did not occur in iCT frogs (Figure 2A). In order to confirm the lack of the expression of this skin peptide in this group, we individually analysed the expression of PPCP by qRT-PCR. Expression of this gene amongst individual iCT frogs was significantly lower than that found among the iWT group (Figure 3B), validating the microarray findings (Figure 2A). Principal component analysis of gene expression in the spleen (Figure 2B) showed that in particular, the iCT 7 day group had the most divergent transcriptional pattern; this was in concordance with the mathematical model where frogs subjected to non-optimal temperatures presented the highest prevalence and intensities of infection. Among the top 50 highly up-regulated genes in the spleen of *Bd* infection in the cold group, seven genes corresponded to serine protease transcripts (trypsinogens, chymotrypsin, zymogen and elastases; Figure 2A). Serine protease proteins are involved in several photolytic pathways but are also recognised effectors of the innate immune system and are associated with generalised inflammation [31], [32]. Our analysis showed a large increase in levels of expression from day 7 to day 42 for these genes (19 fold change; Figure 3B) in iCT animals and a similar, but less pronounced effect, was seen in the iWT frogs (Figure 3B). Here, we speculate that iCT frogs are attempting to compensate for their lack of PPCP-production by upregulating the production of serine proteases as a secondary, inflammatory, innate immune response. The production of serine proteases are pro-inflammatory markers as they represent an increase in mast-cell activity and degranulation [33], [34]; this may well provide a protective role as our prevalence data and modelling show that even the iCT frogs are able to control their *Bd* infections, albeit to a lesser degree than is seen in the iWT groups.

In mammals, inflammatory immune reactions are catalysed by the cross-linking of mast-cells with IgE, and this constitutes the major host-response to mucosal gastrointestinal nematodes [35]. The inverse relationship seen here between PPCP and protease production in animals subjected to cold temperatures suggests that a negative feedback may occur between these two facets of the *S. tropicalis* innate immune response, i.e. that inflammatory responses and secreted AMPs may be mutually inhibitory. If this is the case, then the reduction of AMP-activity seen in iCT and late-stage iWT animals may be the major factor allowing an upregulation of these inflammatory cell-responses.

### Wider Conclusions

We have infected the African Pipid frog *S. tropicalis* at two temperatures and profiled the group prevalence and intensities of infection over 42 days. This has shown that both frogs held at ‘optimal’ temperatures (iWT; 26°C) and those held at ‘non-optimal’ temperatures (iCT; 18°C) are able to survive very high, repeated, doses of *Bd*. Profiling infection using qPCR has shown that both groups of frogs manifest an early peak of infection followed by a reduction in prevalence; this suggests that both groups of frogs are clearing infection however that the frogs held at ‘optimal’ temperatures clear their infections faster. We then asked the question ‘does this effect occur due to either the increased growth of *Bd* at lower temperatures or to more effective host-responses at warmer temperatures?’ We use a simple mathematical model parameterised on measured *Bd* growth rates at different temperatures to address this question. Our findings show that, while the *Bd* growth-rates can explain some of the difference between the prevalence of infection between iCT and iWT groups of frogs, the rate of zoospore clearance needs to be far higher for the iWT animals in order to explain our data, even when taking into account the decrease in *Bd* growth-rates at 26°C (shown as the differences between the ‘blue’ and ‘red’ spots in Figure 1B). This difference in zoospore clearance between the iWT and iCT frogs is shown as the difference *g* in Figure 1A. As the rate of zoospore clearance is analogous to the effectivity of the host response, these findings suggest that frogs held at ‘optimal’ 26°C temperatures are mounting a response that is able to clear *Bd* infections.

We then identified the basis of this host response in ‘optimal’ iWT compared to ‘non-optimal’ iCT frogs by profiling the changes in the host transcriptome for early (day 7) and late (day 42) infections in the major amphibian lymphoid tissue, the spleen. Because we were interested in identifying ecologically-relevant effects, we chose to house animals in groups rather than singly. While this prevents our analysis from looking at individual-level effects (our transcriptional-level responses are for the experimental groups pooled between tanks), our results are ecologically relevant as *S. tropicalis* are colonial in nature and cohabit ponds. Further, a pooling strategy enabled us to extract enough rRNA from these frogs small spleens for direct Cy3/Cy5 labelling; this enabled us to avoid pre-amplifying the RNA using PCR, a process which introduces unknown biases in gene-expression. Recent studies by Kendzidorski *et al*. [36] have shown that most genes in expression studies are not adversely affected by pooling strategies and that the increase in sample-sizes (52 in this study) outweigh the loss of power in ascertaining individual-level-responses.

Our analyses of the *S. tropicalis* spleen-transcriptome showed extensive differences between all of our combinations of experimental groups. Because the frogs spleens were pooled between tanks, we removed tank-level effects. Therefore, the residual changes in gene-expression are attributable to our three major variables, ‘infection’, ‘time’ and ‘temperature’; all of which contribute to major changes in expression levels in the *S. tropicalis* spleen. Despite the complex patterns that we found, we were able to isolate three major signals that discriminated between infected and uninfected animals. These were (1) strong upregulation of innate immune AMP-effectors at host optimal temperatures early in infection, (2) ablation of this response in frogs housed at 18°C and (3) a serine-protease proinflammatory response in both optimal and non-optimally-housed frogs.

One of the great conundrums surrounding the emergence of *Bd* has been its extraordinarily wide host-range in over 350 species, the greatest seen for any vertebrate pathogen to date ([37]; www.spatialepidemiology.net/bd-maps). It has long been suspected that this wide distribution may result from the ability of the pathogen to manipulate and subvert host-immunity or to avoid detection. Recent data by Rosenblum *et al*. [15] have provided important experimental evidence for this hypothesis by showing that several genes that are associated with immune functions, such as toll-like pathogen receptors and complement-pathway genes, are downregulated in infected *S. tropicalis*. Our data also support this hypothesis by showing that putative markers of B-cell, T-cell and complement factors are downregulated in the spleens of animals that have been infected at host-optimal 26°C temperatures. Unfortunately, a full comparison between ours, and Rosenblum *et al*.'s studies is not possible due to the limited gene-set (40%) that are represented on the Operon AROS microarrays that we have used, preventing effective cross-comparisons. However, recent unpublished data targeting a wider panel of adaptive immune-markers in the *S. tropicalis* liver by qPCR confirm our general findings by showing that these markers are all downregulated in infected frogs at host-optimal temperatures (Seidel, Ribas & Fisher, unpub. data). Taken together, these observations stemming from independent studies by different research groups are broadly concordant in their conclusion that there is a generalised down regulation in markers of adaptive immunity in *S. tropicalis* following infection by *Bd*.

Work on another class of eukaryotic pathogens exhibiting wide host ranges, the helminths, has shown that these parasites exhibit widespread subversion of host immunity; strategies include skewing the host response towards a tolerogenic T_H_2 responses, interference with antigen-processing and modulation of antigen-presenting cells [35]. Interestingly, the final two processes are thought to be modulated by nematode homologues of mammalian cystatins, which function to maintain mammalian dendritic cells in their immature state in order to downregulate antigen presentation [38]. In this study we found that the cystastin C precursor peptide was upregulated in iWT frogs when *Bd* burdens were highest (at day 7), suggesting that a modulation of host immunity by this mechanism may be occurring. Searching the *Bd* genome for close homologues of vertebrate proteins with known roles in immune evasion and down-regulation of host immune responses should provide a fruitful approach to identifying whether these specific virulence factors occur. If found, such virulence factors would provide evidence that a long co-evolutionary history has occurred between *Bd* and its Pipid hosts [39], [40]; this is predicted by the ‘Out of Africa’ hypothesis advocated by Weldon *et al*. accounting for the global spread of this pathogen owing to the international trade in Pipid species [41].

It is not clear from our study why markers of adaptive immunity are downregulated in iWT frogs. However, down regulation of *S. tropicalis*' adaptive immunity may be a consequence of the route of infection (cutaneous) and the primary immunity (AMP-mediated and inflammatory innate immunity). The clearest finding from our study was that the most over-expressed transcript (up to x70 fold) was associated with an innate-effector AMP. The various arms of the vertebrate immune response are interdependent, and the upregulation of one arm has extensive effects on the expression of other immune modes; the T_H_1/T_H_2 inhibitory dicotomy is a well known example of this effect. It may be that over-expression of AMPs has a dampening effect on other limbs of the amphibian immune-system, resulting in under-expression of adaptive-immune effectors. However, it is perhaps naïve to expect that the effects that we are describing here can be captured by simple ‘innate versus adaptive’ models, and a systematic network analysis of *S. tropicalis* interactomes on an individual basis is required to dissect these interactions [42]. Recently reported preliminary results demonstrating the development of high-titer antibodies to *Bd* in *Xenopus laevis* injected with the heat-killed pathogen and subsequently exposed to live zoospores [43], suggest that Pipids are able to make anti-*Bd* antibodies. In this case, then the route of infection is likely to be important in determining what sort of host response is manifested. Importantly, our data show that, owing to the lack of a strong adaptive immune response but high innate response, it is unlikely that *S. tropicalis* can be primed to respond to subsequent challenges of *Bd* by using a cutaneous method of infection; we suggest that it will not be easy to develop vaccines against *Bd* and it is unlikely that wild populations will acquire a natural immunity to the pathogen.

Mathematical modelling of the dynamics of infection in our experimental treatments has shown that frogs maintained below their temperature optimum reduce the generation of a successful anti-*Bd* response, resulting in higher infectious burdens. Analysis of the iCT versus control CT and iWT animals suggests that this may be due to a lack of AMP production; there was no expression of PPCP in the iCT frogs. This may explain why Rosenblum *et al*. found no evidence of AMP upregulation in their whole-genome arrays as, in their experimental design, all frogs were housed at 18°C; these conditions correspond to our iCT group which was not found to express markers of AMP activity. These findings are likely to be ecologically relevant as it has been shown that, in the field, immunity is temperature-dependent and forced by seasonal patterns. For example seasonal surveys of adult Red spotted newts (*Notophthalmus viridescens*) by Raffel *et al* [12] showed a spring lag effect in lymphocyte levels and strong seasonal acclimatization of lymphocyte, neutrophil and eosinophil levels in the autumn. A body of work now exists on the prerequisite of a successful AMP-response to manifest resistance to chytridiomycosis at the species level [18], [44], [45]. Taken together, these studies suggest that factors that influence AMP production are likely to change whether populations/species are refractory to chytridiomycosis, or whether they succumb. It is important to note that there is an apparent association between declines of species and altitude, with populations at higher altitudes suffering greater losses and extinction rates [6], [46]. There is now a clear need for such field-based immunological studies among infected populations to investigate the importance of the host response in the epidemiological triad (host, *Bd* and the environment). Such studies will quantify the influence that changing climates are able to exert on the ectothermic host response to this devastating emerging pathogen.

## Materials and Methods

### Ethics Statement

All experiments involving amphibians were conducted under Licence from the British Home Office following the Animals (Scientific Procedures) Act of 1986. Welfare of the amphibians followed the principles of the 3R's; reduction, refinement and replacement and were overseen by a named Veterinarian as well as Animal Care and Welfare Officers. All experiments were preceded by full ethical review by the Imperial College Ethical Review Committee and the British Home Office, and were fully approved by these two organisations.

### *Silurana tropicalis* Infection and Temperature Model

52 adult female frogs of the species *S. tropicalis* (average weight 12.6 g) were reared in 8 water tanks (3–4 frogs per tank) in temperature-controlled rooms stabilised at 18°C, cold temperature group (CT) or 26°C warm temperature group (WT); Figure S1. Frogs were infected with *Bd* by individual exposures to 10^6^ *Bd* zoospores in a 100 ml bath for 3 hours (these groups are designated iCT or iWT). *Bd* strain IA042 passage 10 was isolated from infected *Alytes obstetricans* in the Pyrenees, Spain and cultured in ThGL liquid media; zoospore inocula were standardised by counting motile zoospores using a haemocytometer. Isolate IA042 is known to have high virulence from previous experiments [39]. Exposures were repeated twice a week for 7 or 42 days (iCT7, iCT42, iWT7, iWT42). Control animals were similarly treated with regular transfer to an experimental bath, but were exposed to ThGL medium as a sham-infection (groups CT7, CT42, WT7, WT42). Animal welfare was checked daily while feeding and equal amounts of food given to all groups. We observed no sign of illness beyond skin-sloughing early on in the experimental regime, and no mortality occurred. Throughout the experiment, dermal swabs were taken (days 0, 7, 26 and 42) from each animal and infection monitored by a *Bd*-specific TaqMan Assay (Boyle *et al*. 2004) (Figure S1). In order to ensure that we were working with a *Bd*–free colony, frogs were swabbed and tested prior to starting the experiment and were all found to be negative. At days 7 and 42, animals were euthanized by ten-minute exposure to an overdose of MS-222 before the spleen from each animal was rapidly dissected on ice and placed into RNAlater (Qiagen), then kept at −80°C until further transcriptomic analysis (microarray & qRT-PCR).

### *Bd* Dynamics and Mathematic Modelling

The prevalence and intensities of infection were estimated using a qPCR Taqman assay [47]. Briefly, *Bd* DNA was extracted from swabs and a TaqMan-probe assay used in an Applied Biosystems 7300 Sequence Detection System. Samples were prepared in duplicate with negative controls, and a standard curve was used to quantify the number of *Bd* zoospore genomic equivalents. The observed prevalence of infection are shown in Figure 1A.

In order to dissect the relative importance of host immunity versus *Bd* growth-rates, a mathematical model of the experiment was created assuming a constant rate of clearance of infection, and infection only at infection procedures. The model splits the experimental animals into three categories: Susceptible (S), Infected (I) and Recovered (R). All animals begin in the Susceptible category, from which they move into the Infected category at *β*S animals per infection procedure (which occurred bi-weekly). Infected animals then recover into the Recovered category at rate γI (such that the time spent infected is exponentially distributed.). The equations for the model are given below.
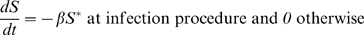
(1)

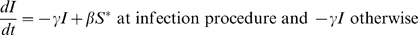
(2)

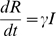
(3)


where *β* is the rate of infection per procedure, *γ* is the recovery rate once infected, and * indicates a change at a single time point.

This model makes two major assumptions: that the chance of infection via transmission from an infected animal is negligible compared to the chance of being infected by a procedure; and that once an animal recovers it now has either, or a combination of, such a low chance of infection that it will now no longer be infected, or such a fast rate of recovery that if infected it will clear zoospores fast enough that it will show up as negative when tested for infection – i.e. once recovered, animals will stay in the Recovered category. Transmission is assumed to be negligible compared to infection by procedure as the highest intensity of infection reported from any animal was 15.12 zoospore genomic equivalents, whereas each procedure exposed the animals to 10^6^ zoospores, an amount several magnitudes greater than any possible exposure from other animals. To test whether reinfection can be assumed to be negligible, a model was created in which animals in the Recovered compartment were allowed to become reinfected, with different infection and recovery rates to that of newly infected animals. Fitting this model to the prevalence data gave a greatly reduced rate of infection and increased rate of recovery for re-infected recovered animals, supporting the assumption.

The average infected lifespan (*T*) for both iCT (infected 18°C) and iWT (infected 26°C) animals was calculated from the infection model as the inverse of γ. In order to discern whether differences in infected lifespan between the two models can be explained entirely by differences in *Bd* growth-rate, or whether a differing host response at each temperature was required, the host response necessary at each temperature in order to return the zoospore count to 0.1 genome-equivalents (the amount above which infection is indicated) at the end of the average infected lifespan was then calculated. This was done by assuming *Bd* growth within the host is exponential, with a rate determined by two opposing factors: the intrinsic exponential growth of *Bd* at that temperature (*r*), and the rate of zoospore clearing by the host (*c*). To determine *r* the daily exponential growth rate of *Bd* in culture was calculated for each temperature [48] and then fitted to a third order polynomial equation (Figure 1B) from which *r* was calculated for both experimental temperatures. As so little is known about the immune response of *S. tropicalis* to fungal infections, the simplest form possible for the rate of clearing zoospores has been assumed, with *c* remaining constant and acting from the moment of infection. Provided that the exponential growth rate of *Bd* in culture is comparable to the uninhibited growth rate of *Bd* on the host, and that further infection procedures have no effect on the number of zoospores infecting the host, the required rate of clearing can then be calculated using the equation *Ze^(r−c)T^* = 0.1, where *Z* is the number of zoospores initially infecting the host, and has been assumed to be directly proportional to the rate of infection and the number of zoospores used per procedure. The values of *c* for both warm and cold experiments were then compared. Mathematical models were fitted to infection dynamics using Berkeley Madonna 8.3.14.

### Microarray Hybridization and Transcriptomic Analysis

RNA samples from all spleen tissues were individually prepared (n = 52) using Qiagen RNeasy Mini Kit Columns (Qiagen) and further treated with DNaseI (Invitrogen). RNA quality and quantity was assessed using Bioanalyzer 2100 with RNA 6000 Nano LabChip Kit (Agilent Technologies) and NanoDrop 1000 Spectrophotometer (NanoDrop Technologies). To achieve enough RNA for direct Cy5 labelling without resorting to PCR pre-amplification, RNA spleens from 2 or 3 animals were pooled to give 3 pools of spleen RNA samples for each group of animals (see Figure S1) following published studies [36]. Samples were pooled between tanks but within experimental groups to remove tank-level effects. Sample (4 µg) of each RNA-pool were labelled using SuperScript III cDNA synthesis kit, oligo d(T) primer (Invitrogen) and Cy5-dCTP (GE Healthcare/Amersham). The resulting products were purified using a QIAquick cleaning kit (Qiagen). In parallel, a universal reference RNA sample was created from the spleen of an outgroup of animals (n = 4), which was further amplified to achieve large quantities using Amino Allyl MessageAmp™ II aRNA Amplification (Ambion). The resulting aRNA was labelled using a Cy3 mono-Reactive Dye Pack (GE Healthcare/Amersham). This Cy3-labelled aRNA was served as an universal reference (see below) to provide consistent slide-to-slide and experiment-to-experiment comparability. The principle and reliability of universal reference system using pooled RNA or amplified RNA were originally described [49], [50].

A total of 24 *Xenopus tropicalis* V1.0 AROS DNA microarray slides were obtained from Operon Biotechnologies (www.operon.com) and previously described [51]. The array design is available at ArrayExpress (www.ebi.ac.uk) under accession number A-MEXP-1446. Slide hybridization and washing steps were performed according to manufacture's protocol using buffers provided with the kit (Operon Biotechnologies). Each array slide was co-hybridized with one of the Cy5 labelled experimental samples and Cy3 labelled aRNA (universal reference). The resulting slides were scanned using a GenePix 4000B (Axon) microarray scanner and the scanned images were analysed with GenePix Pro6.0 (Axon). Statistical analysis was carried out using Genespring GX 7.3 (Agilent). Background-subtracted spot intensities resulting from hybridisation of Cy5-labeled cDNA derived from experimental RNA (signal channel) were divided by the intensity of the signal derived from Cy3-labeled aRNA (control channel). Log-transformed ratios were normalized by applying the intensity-dependent data analysis technique LOWESS, using 20% of the data for smoothing. Only genes not marked absent by GenePix Pro analysis were included. If the value for the reference channel was less than 10 then a value of 10 was used instead. Data arising from the three pooled biological replicates were combined as follows:

For each gene, the normalized log-transformed ratio arising from samples harvested from the infection group kept at 26°C for 7 days (iWT7) was re-normalized to the median value, as was that arising from uninfected group kept at 26°C for 7 days (cWT7). These were combined to give a ratio iWT7/cWT7. Likewise, the ratio were also generated for iWT42/cWT42, iCT7/cCT7 and iCT42/cCT42, representing experimental groups conditioned at 26°C for 7 days, at 18°C for 7 days and 18°C for 42 days, respectively. Differentially expressed genes were identified as genes showing more than 1.5-fold up- or down-regulation (*p≤*0.01) using a Student *t*-test in GeneSpring. Fully annotated microarray data have been deposited under ArrayExpress accession number E-MEXP-1936. Expression values are shown in Table S1.

### Microarray Validation

Microarray-based gene expression patterns were confirmed for a subset of 10 genes (Figure 3A, Table S2) with known or suspected roles in immunity, by using qRT-PCR. In order to maximise the power of our data, individual analyses were run for each of the experimental frogs. A total of 52 individual spleen RNA samples, 12 for control (CT, WT) and 14 for infected animals (iCT, iWT) for each treatment group (7 and 42 days), were reverse-transcribed using SuperScript III and oligo d(T) (Invitrogen). The resulting cDNA were used as templates in qRT-PCR run in Applied Biosystem 7100 as following: Cycle 1; 50°C 2 min., Cycle 2; 95°C 10 min., Cycle 3; 95°C 15 sec., 60°C 1 min for 40 times. Samples were run as triplicates, with water and RNA as negative controls. All expression values were normalised against the constitutively-expressed gene, elongation factor α. Relative gene expression levels for these qRT-PCR results were analysed using the 2^−ΔΔ*C*T^ method [52] showing log_2_ Relative Quantification versus the control (iWT7/cWT7, iWT42/cWT42, iCT7/cCT7 and iCT42/cCT42) ± Standard Error.

Further gene expression analysis was performed in order to assess the reliability of the transcriptomics results, for two target genes of interest; the skin peptide PPCP and the serine-protease trypsinogen 2. These two genes were individually tested by qRT-PCR from the spleen RNA all 52 experimental frogs prior to pooling. Statistical analyses of the Relative Quantification results were completed using the Student *t*-test in the *R*-Software Package.

The subset of validated genes were (Table S2): muscle specific gene (Xt_10001800), trypsinogen 2 (Xt_10000537), T-cell (Xt_10005209), CD82 antigen (Xt_10002930), TRAF (Xt_10007227), Preprocaerulein type III precursor (Xt_10003079), epithelial membrane protein 2 (Xt_10006495), cystatin C precursor (Xt_10001311), BCL2/adenovirus (Xt_10007462) and protein EST EC2BBA27CH07 (Xt_10007462).

## Supporting Information

Figure S1 Schematic of the experimental design. Frogs were infected with *Bd* by individual exposures to 106 *Bd* zoospores in a 100 ml bath for 3 hours (iCT or iWT). These exposures were repeated twice a week for 7 or 42 days (iCT7, iCT42, iWT7, iWT42). Control animals were similarly treated with regular transfer to an experimental bath, but exposed to ThGL medium as a sham-infection (CT7, CT42, WT7, WT42). Throughout the experiment, dermal swabs were collected from each animal and infection monitored by *Bd*-specific TaqMan Assay (*). For transcriptomic analysis RNA was extracted individually from spleen tissues. To achieve enough RNA samples for direct Cy5 labelling without amplification for microarray hybridization, spleen RNA from 2 or 3 animals were pooled to give 3 pools of spleen RNA samples for each animal group. In parallel, a universal reference RNA sample was created from spleen of an out-group animals (n = 4), and was largely amplified aRNA. 3 and 4 microarray slides were used for each experimental group for control and infected treatment respectively (a total of 24 microarray slides). Microarray validation and other transcriptomic analysis were performed by qRT-PCR individually for each animal group of the experiment.
(0.55 MB TIF)

Table S1 Expression values of the microarray results in spleen of animals *Bd* infected after 7 or 42 days. For statistical analysis, see the Materials and Methods section of the manuscript. Fully annotated microarray data have been deposited under ArrayExpress with accession number E-MEXP-1936.
(0.18 MB XLS)

Table S2 Primer information for the genes used in validating the microarray analysis.
(0.05 MB PDF)
