# Supplementary figures and images for: Expression Profiling the Temperature-Dependent Amphibian Response to Infection by Batrachochytrium dendrobatidis
Source: PLoS One. 2009 Dec 22;4(12):e8408. doi: 10.1371/journal.pone.0008408 (PMC2794374; doi:10.1371/journal.pone.0008408)

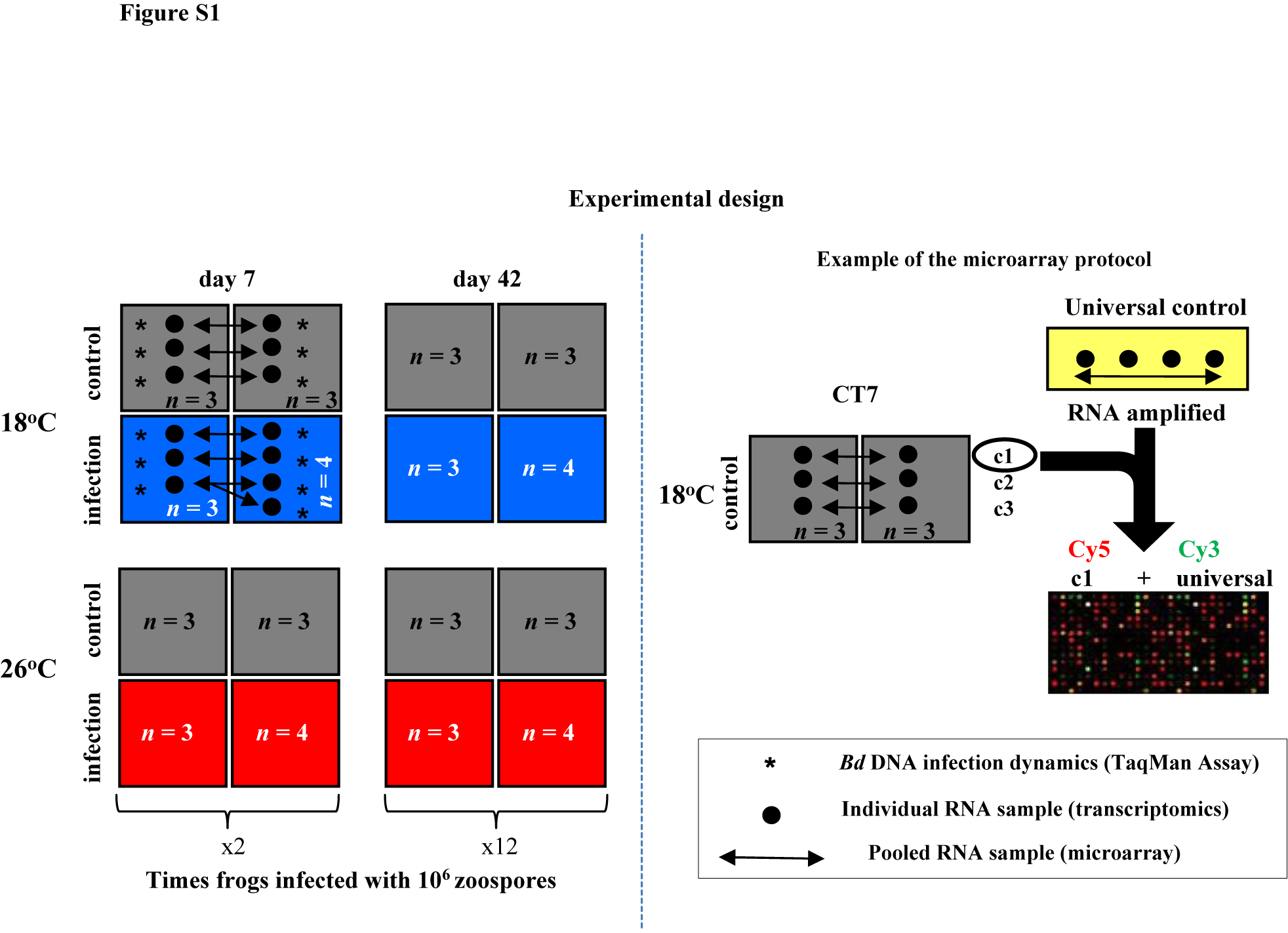

Supplement: Figure S1 — Schematic of the experimental design. Frogs were infected with Bd by individual exposures to 106 Bd zoospores in a 100 ml bath for 3 hours (iCT or iWT). These exposures were repeated twice a week for 7 or 42 days (iCT7, iCT42, iWT7, iWT42). Control animals were similarly treated with regular transfer to an experimental bath, but exposed to ThGL medium as a sham-infection (CT7, CT42, WT7, WT42). Throughout the experiment, dermal swabs were collected from each animal and infection monitored by Bd-specific TaqMan Assay (*). For transcriptomic analysis RNA was extracted individually from spleen tissues. To achieve enough RNA samples for direct Cy5 labelling without amplification for microarray hybridization, spleen RNA from 2 or 3 animals were pooled to give 3 pools of spleen RNA samples for each animal group. In parallel, a universal reference RNA sample was created from spleen of an out-group animals (n = 4), and was largely amplified aRNA. 3 and 4 microarray slides were used for each experimental group for control and infected treatment respectively (a total of 24 microarray slides). Microarray validation and other transcriptomic analysis were performed by qRT-PCR individually for each animal group of the experiment. (0.55 MB TIF) [file pone.0008408.s001.tif]
