# Supplementary material for: Expression Profiling the Temperature-Dependent Amphibian Response to Infection by Batrachochytrium dendrobatidis
Source: PLoS One. 2009 Dec 22;4(12):e8408. doi: 10.1371/journal.pone.0008408 (PMC2794374; doi:10.1371/journal.pone.0008408)
Supplement: Table S2 — Primer information for the genes used in validating the microarray analysis. (0.05 MB PDF) [file pone.0008408.s003.pdf]

Microarray Validation Genes		
**Primer Name**	**Accession number**	**Sequence 5'-3'**
epithelial membrane protein	Xt_10003079	For ATGCCTGTGTGTCATGATTGCTGC
		Rev TAGTGTGAAGGCAAAGGCGATCCA
trypsinogen 2	Xt_10006365	For AATGAACATTGGGTGGTGTCTGCC
		Rev TGGCGAATGATCTTGGCAGATTGG
PPCP	Xt_10007227	For GCCCGAAATTTGCTGGGATCACTT
		Rev TGCATTAGGTCCATCAGCAAAGCG
muscle specific gene	Xt_10001800	For TCTGGCAAGCACTGGATGAGAACT
		Rev ACCGGTCAATGTGCTGGTAAGTGA
Unknown protein	Xt_10007462	For AACCTGTCTTTACTTTCCGCCGCA
		Rev AGAGGGAGCATGGAATGCCAGTTT
TRAF	Xt_10002930	For AGGTAAAGCCGGAGCTGATGAAGA
		Rev TCCTCCCAAACCGTCAAATCCAGT
T-cell	Xt_10000537	For TTCCTCTTTGCTCTCTGGCTGGTT
		Rev TGACAGCTGGTCGACTGAAACACT
CD82 antigen	Xt_10005209	For ACTGTGGTCCAGAACTCCACCAAT
		Rev ATTGTATTGGCAGAAGCCGTTGGG
cystatin C precursor	Xt_10006495	For AGCACAGCCTACTAGAAGGAACCA
		Rev TGCCTGGAGGAACATCCAAGAACT
BCL2/adenovirus	Xt_10001311	For TCAGCATGAGGAACACCAGTGTCA
		Rev TTCCGATGTAGACTCCCAGTCCAA
Ef-α	BC157768	For CCTGAATCACCCAGGCCAGATTGGTG
		Rev GAGGGTAGTCTGAGAAGCTCTCCACG
